# Transgenic conversion of ω-6 to ω-3 polyunsaturated fatty acids via *fat-1* reduces the severity of post-traumatic osteoarthritis

**DOI:** 10.1186/s13075-020-02170-7

**Published:** 2020-04-15

**Authors:** Kelly A. Kimmerling, Sara J. Oswald, Janet L. Huebner, Dianne Little, Virginia B. Kraus, Jing X. Kang, Chia-Lung Wu, Farshid Guilak

**Affiliations:** 1 Department of Orthopaedic Surgery, Washington University in St. Louis, Campus Box 8233, Couch Biomedical Research Bldg, Room 3121, St. Louis, MO 63110 USA; 2 Shriners Hospitals for Children - St. Louis, St. Louis, MO USA; 3 Duke Molecular Physiology Institute, Duke University School of Medicine, Durham, NC USA; 4 Department of Basic Medical Sciences, Purdue University, West Lafayette, IN USA; 5 Department of Biomedical Engineering, Purdue University, West Lafayette, IN USA; 6 Department of Medicine, Duke University School of Medicine, Durham, NC USA; 7 Laboratory for Lipid Medicine and Technology, Department of Medicine, Massachusetts General Hospital and Harvard Medical School, Boston, MA USA

**Keywords:** Metabolic syndrome, Diabetes, Adipocytokine, Cartilage, Arthritis

## Abstract

**Background:**

Dietary fatty acid (FA) content has been shown to influence the development of post-traumatic osteoarthritis (PTOA) in obesity. We used the *fat-1* transgenic mouse to examine the hypothesis that endogenous reduction of ω-6 to ω-3 FA ratio, under the same dietary conditions, would mitigate metabolic inflammation and the pathogenesis of PTOA in obese male and female mice.

**Methods:**

Male and female *fat-1* and wild-type littermates were fed either a control diet or an ω-6 FA-rich high-fat diet and underwent destabilization of the medial meniscus (DMM) surgery to induce PTOA. OA severity, synovitis, and osteophyte formation were determined histologically, while biomarker and lipidomic analyses were performed to evaluate levels of adipokines, insulin, pro-/anti-inflammatory cytokines, and FAs in serum and joint synovial fluid. Multivariable models were performed to elucidate the associations of dietary, metabolic, and mechanical factors with PTOA.

**Results:**

We found that elevated serum levels of ω-3 FAs in *fat-1* mice as compared to wild-type controls fed the same diet resulted in reduced OA and synovitis in a sex- and diet-dependent manner, despite comparable body weights. The *fat-1* mice showed trends toward decreased serum pro-inflammatory cytokines and increased anti-inflammatory cytokines. Multivariable analysis for variables predicting OA severity in mice resulted in correlations with serum FA levels, but not with body weight.

**Conclusions:**

This study provides further evidence that circulating FA composition and systemic metabolic inflammation, rather than body weight, may be the major risk factor for obesity-associated OA. We also demonstrate the potential genetic use of ω-3 FA desaturase in mitigating PTOA in obese patients following injury.

## Introduction

Post-traumatic osteoarthritis (PTOA) is a debilitating joint disease that develops following trauma to the joint [1]. It is estimated that of the 27 million patients with osteoarthritis (OA), approximately 12% are due to trauma. Currently, there are no disease-modifying drugs available for this condition. While the main risk factors for OA include joint injury, age, sex, and genetics, the primary preventable risk factors for this disease are obesity and obesity-associated effects such as metabolic syndrome [2, 3].

The mechanisms relating diet-induced obesity and OA are not fully understood [4], but may involve several factors such as altered joint loading, systemic inflammation, and/or dietary fatty acid (FA) content. Obesity-associated inflammation is characterized by elevated serum levels of pro-inflammatory cytokines such as interleukin-1 (IL-1), IL-6, and tumor necrosis factor alpha (TNF-α) [5], as well as altered levels of adipokines such as leptin and adiponectin [6]. Furthermore, these same cytokines are also upregulated following joint injury and with PTOA [7–10]. In humans, switching to an ω-3 FA-rich diet reduces the serum levels of IL-1β and TNF-α, and increasing evidence suggests that dietary supplementation with ω-3 polyunsaturated fatty acids (PUFAs) can ameliorate metabolic syndrome and modulate circulating levels of leptin and adiponectin [11–13]. These data provide the rationale for this study aimed at genetically altering the ratio of ω-6 FAs to ω-3 FAs via an ω-3 FA desaturase in the context of a PTOA model system.

The *fat-1* gene encodes an ω-3 FA desaturase that was originally identified in *Caenorhabditis elegans* and is not found in mammalian cells. This desaturase dehydrogenates ω-6 FAs to ω-3 FAs by adding a double bond at the third carbon from the methyl terminus [14]. Kang et al. produced a transgenic mouse model that constitutively expresses the *fat-1* gene–encoded ω-3 FA desaturase throughout the body of the animal. Conventionally, to investigate the effect of dietary FAs on disease progression, animals need to be fed different diets with specific FA compositions. The *fat-1* transgenic mouse provides a unique model to study the role of ω-3 FAs in OA pathogenesis without the confounding factors introduced by diet intervention because both wild-type (WT) and *fat-1* animals can be fed the same diet, relying on ω-3 FA desaturase to increase endogenous ω-3 FA levels. Recently, Cai et al. showed that *fat-1* mice and WT littermates exhibited comparable idiopathic OA scores when fed a lean diet rich in ω-6 FAs [15]. In a PTOA model, female *fat-1* mice showed reduced OA scores and increased levels of ω-3 FAs in the serum and cartilage compared to the WT mice [16]. Although both studies provide important insights into how the ω-6/ω-3 FA ratio may affect the development of PTOA in lean animals, the influence of endogenous ω-3 FAs on PTOA progression in obesity and the effects of sex as a variable remain unknown.

In this study, we hypothesized that obese *fat*-*1* mice fed a diet rich in ω-6 FAs will exhibit reduced PTOA-related joint degeneration compared to WT mice in a sex-dependent manner. Biomarker and lipidomic analyses were also performed to evaluate levels of adipokines, insulin, cytokines, and FAs in serum and joint synovial fluid.

## Methods

Detailed methods are provided in online supplementary documents.

### Animals

**Fig. 1:**
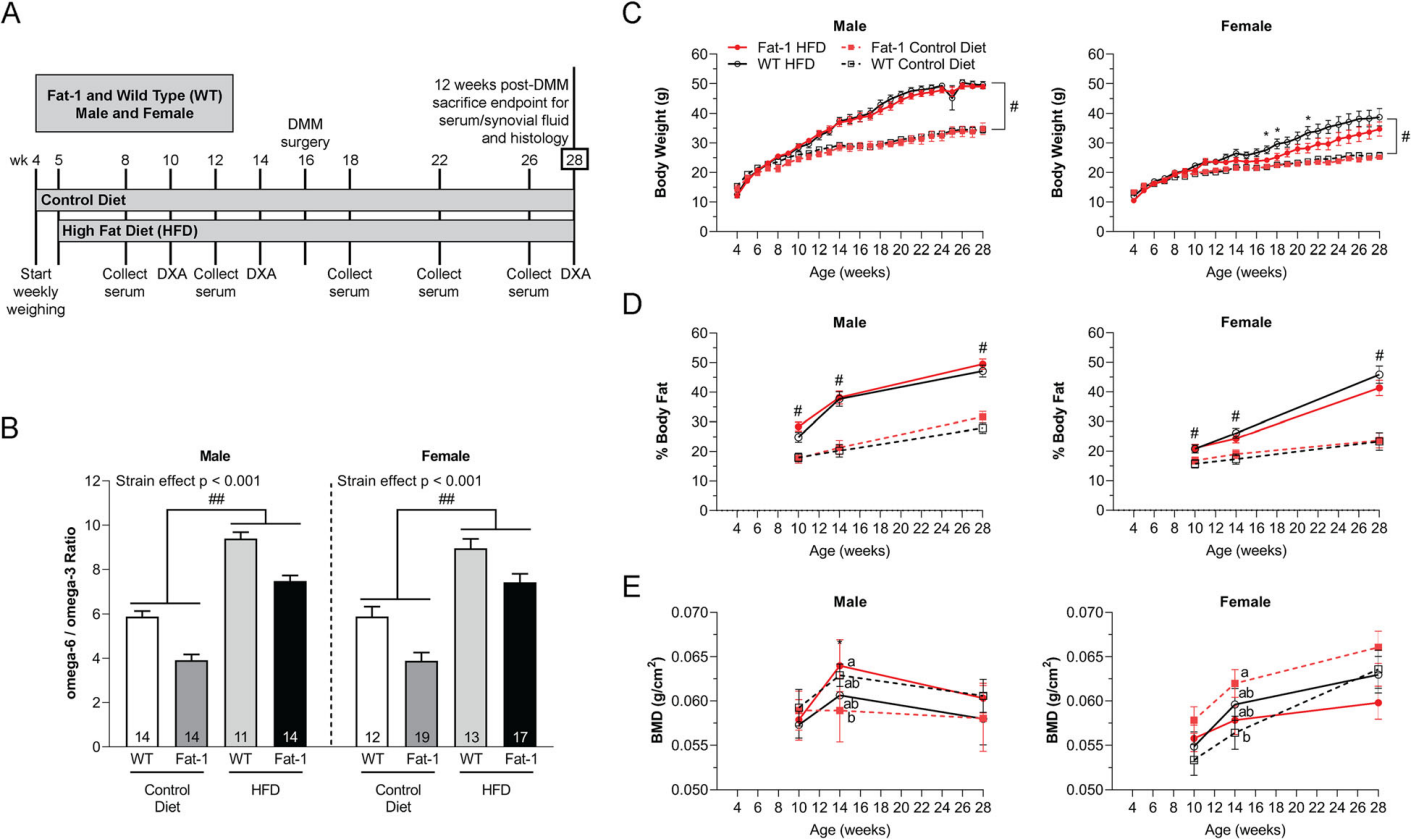
**a** The experimental design. Control and HFD treatment started at 5 weeks of age for both WT and *fat-1* mice. DMM was performed at 16 weeks of age, and animals were sacrificed 12 weeks post-DMM surgery to evaluate OA and joint synovitis. **b** Serum ω-6/ω-3 FA ratio. Two-way ANOVA within the same sex. *p* < 0.001, WT vs. *fat-1* strain effect. ^##^*p* < 0.001, HFD vs. control diet. The number (*n*) of the mice used in each group is indicated on plots. Data presented as mean ± SEM. **c** Body weight and **d** percent body fat over time. Two-way repeated measures ANOVA followed by Fisher’s LSD post hoc. ^#^*p* < 0.05, HFD vs. control diet. *p < 0.05, WT female mice treated with HFD vs. *fat-1* female mice treated with HFD. Data presented as mean ± SEM. **e** Whole-body BMD. Two-way repeated measures ANOVA followed by Fisher’s LSD post hoc. Groups not sharing the same letter are significantly different, *p* < 0.05, at 14 weeks of age. Data presented as mean ± SEM. For BMD and percent fat: male control diet WT *n* = 16, *fat-1 n* = 13; male HFD WT *n* = 12, *fat-1 n* = 15; female control diet WT *n* = 12, *fat-1 n* = 17; and female HFD WT *n* = 13, *fat-1 n* = 16. For body weight: male control diet WT *n* = 16, *fat-1 n* = 8; male HFD WT *n* = 7, *fat-1 n* = 15; female control diet WT *n* = 12, *fat-1 n* = 17; and female HFD WT *n* = 8, *fat-1 n* = 11

All animal procedures were approved by the Duke University Institutional Animal Care and Use Committee. *fat-1* mice and littermate wild-type (WT) controls were bred for this study. Mice were placed on either a control diet (“lean” group) or an ω-6 FA-rich high-fat diet (HFD or “obese” group) starting at 5 weeks of age (Fig. 1a). Dual-energy X-ray absorptiometry (DXA) was used to assess bone mineral density (BMD) and percent body fat at 10, 14, and 28 weeks of age.

### Destabilization of the medial meniscus (DMM) model

At 16 weeks of age, mice underwent DMM surgery in the left hind limb to induce PTOA [17]. The right hind limb remained non-operated as a contralateral control. At sacrifice 12 weeks post-DMM, both hind limbs from each mouse were harvested and processed into sections for the assessment of articular cartilage degeneration, synovitis, and osteophyte formation.

### Serum and synovial fluid biomarker assays

Serial serum was collected at 8, 12, 18, 22, and 26 weeks of age. At sacrifice 12 weeks post-DMM, serum and synovial fluid were collected for biomarker and lipidomic analyses.

### Statistical analysis

All results were analyzed using the SPSS Software Package (IBM, Armonk NY, USA), with significance reported at the 95% confidence level.

## Results

### Decreased serum ω-6/ω-3 FA ratio in *fat-1* mice compared to WT mice

High-fat feeding of both male and female mice increased the serum ω-6/ω-3 FA ratio (Fig. 1b) at 28 weeks of age. However, this ratio was significantly decreased in *fat-1* mice compared to WT mice. Indeed, *fat-1* mice, independent of sex and diet treatments, demonstrated lower serum ω-6 FA levels (although the statistical significance was only observed in male mice fed the control diet), suggesting endogenous conversion of ω-6 FAs into ω-3 FAs (see supplementary Fig. S1A-B). Additionally, both WT and *fat-1* mice also exhibited similar serum levels of saturated fatty acids (SFAs) and monounsaturated fatty acids (MUFAs) (see supplementary Fig. S1C-D).

### Comparable body weight and percent body fat in *fat-1* and WT mice under the same diet

Mice fed the HFD exhibited increased body weight in comparison with the mice treated with the control diet (Fig. 1c). In addition, male mice had relatively higher body weight than female mice regardless of the strain or diet treatment. However, independent of sex and diet, *fat-1* mice had similar body weights to those of the WT mice under the same diet at 28 weeks of age; in contrast, obese *fat-1* female mice had significantly decreased body weight compared to obese WT female mice at 17, 18, and 21 weeks of age.

At all measured time points, independent of sex, obese mice had significantly increased percent body fat compared to the mice treated with the control diet (Fig. 1d). Both male and female *fat-1* mice had percent body fat comparable to WT mice of the same sex under the same diet treatment.

### Sex- and diet-dependent effect of decreased serum ω-6/ω-3 FA ratio on whole-body BMD

Male *fat-1* mice fed the control diet had lower whole-body bone mineral density (BMD) compared to male WT mice under the same diet at 14 and 28 weeks of age (Fig. 1e). Once again, this trend was opposite for females; the female *fat-1* mice fed the control diet had significantly higher whole-body BMD compared to female WT mice under the same diet at 14 weeks of age.

Obese male *fat-1* mice had a trend toward increased whole-body BMD compared to male obese WT mice at 14 and 28 weeks of age (Fig. 1e). This trend once again was opposite for females, as female obese *fat-1* mice trended toward having lower whole-body BMD compared to obese WT mice at 14 and 28 weeks of age.

### Reduced injury-induced OA in obese *fat-1* mice in a sex-dependent and diet-dependent manner

**Fig. 2:**
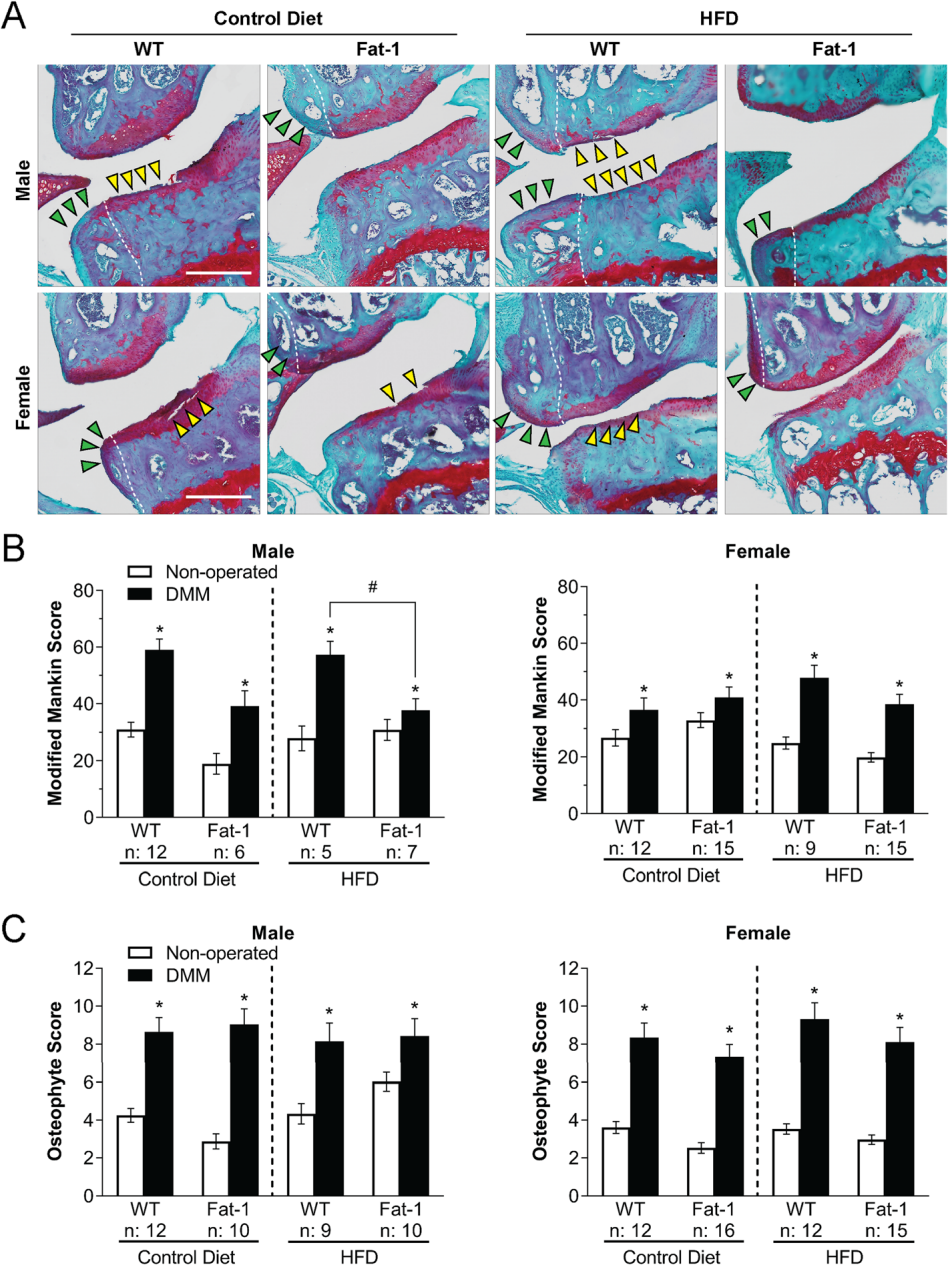
**a** Safranin-O– and Fast Green–stained DMM-operated joints. Yellow arrowheads: severe cartilage loss and damage. Green arrowheads: osteophytes. White dashed lines: boundary between bone and osteophyte. Scale bar = 1 mm. **b** Modified Mankin score and **c** osteophyte scores for total joints. Two-way repeated measures ANOVA within the same sex followed by Fisher’s LSD post hoc. *p < 0.05, DMM-operated vs. non-operated joints. ^#^*p* < 0.05, HFD *fat-1* vs. WT DMM-operated joints. The number (*n*) of the mice used in each group is indicated on plots. Data presented as mean ± SEM

DMM surgery resulted in a significant joint degeneration at 12 weeks post-surgery (Fig. 2a, b). OA scores were higher in WT male compared to those in WT female mice. Despite obesity, male and female *fat-1* mice exhibited decreased OA in the DMM-operated joints as compared to obese WT littermates, although statistical significance was only observed in male mice (Fig. 2b). Lean male *fat-1* mice fed the control diet, but not lean female *fat-1* mice, also showed a similar trend toward lower OA scores in the DMM-operated joints versus their corresponding lean WT littermates.

### Similar osteophyte scores in *fat-1* mice compared to their WT littermates

DMM surgery led to osteophyte formation in the operated joints (Fig. 2c). However, *fat-1* mice, regardless of sex or diet, did not show altered osteophyte scores compared to their corresponding WT littermates.

### Reduced synovitis trends for *fat-1* mice in a sex-dependent manner

**Fig. 3:**
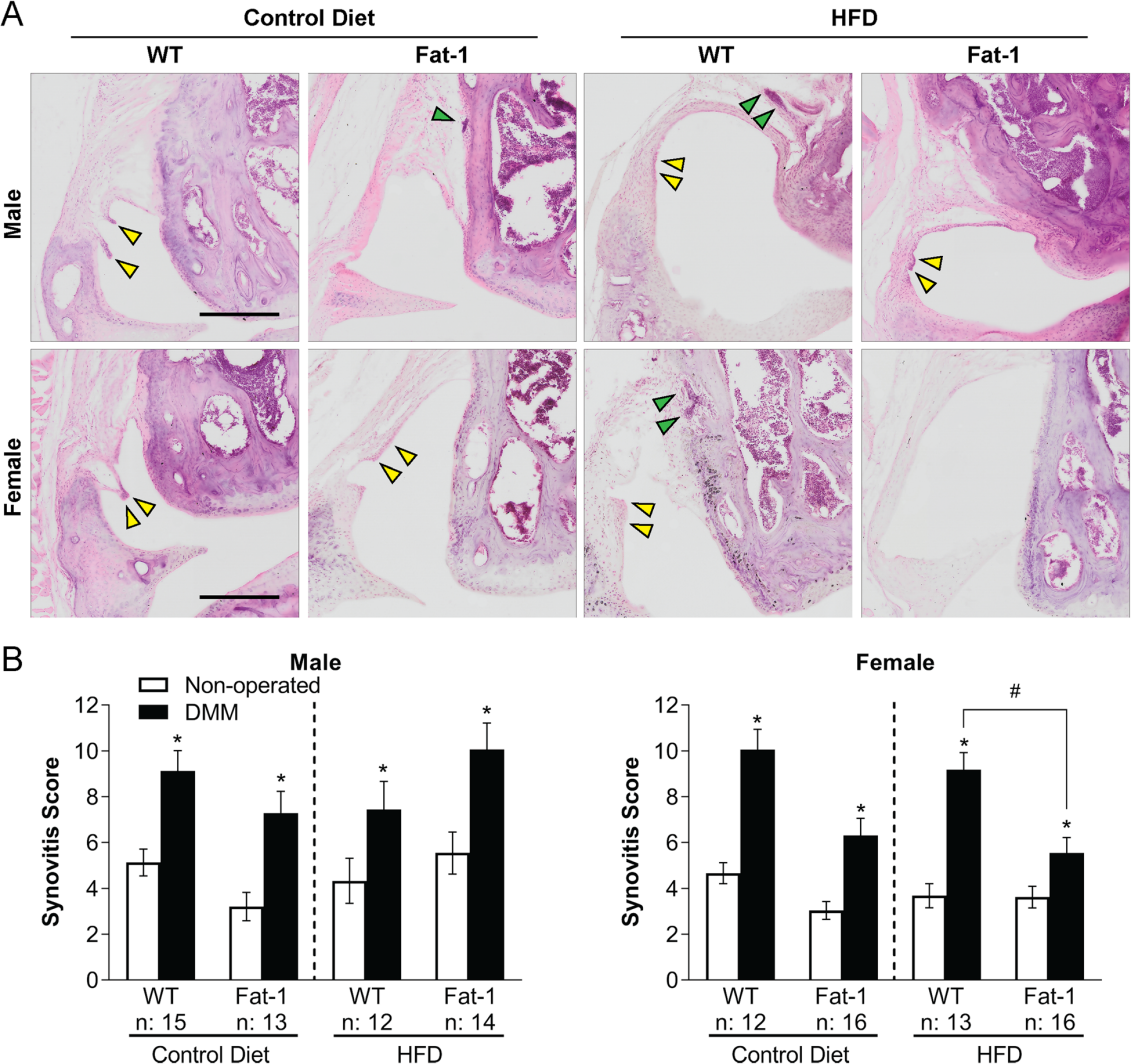
**a** Hematoxylin and eosin (H&E) staining of the medial femoral condyle in DMM-operated joints. Yellow arrowheads: formation of pannus and infiltration of cells in the synovial layer. Green arrowheads: inflammatory aggregates of immune cells in the synovium. Scale bar = 250 μm. **b** Total joint synovitis score. Two-way repeated measures ANOVA within the same sex followed by Fisher’s LSD post hoc. *p < 0.05, DMM-operated vs. non-operated joints. ^#^*p* < 0.05, HFD *fat-1* vs. WT DMM-operated joints. The number (*n*) of the mice used in each group is indicated on plots. Data presented as mean ± SEM

In all groups, DMM surgery increased joint synovial inflammation in mice, accounting for diet treatment, strain, and sex (Fig. 3a, b). Obese female *fat-1* mice on the HFD had statistically significant decreased synovitis scores in the DMM-operated joints compared to obese female WT mice, with control diet female mice showing a similar trend. Male *fat-1* mice did not have attenuated synovial inflammation in the DMM-operated joints regardless of strain or diet treatments.

### Obesity and decreased ω-6/ω-3 FA ratio affected serum insulin and leptin levels in a sex-dependent manner

**Fig. 4:**
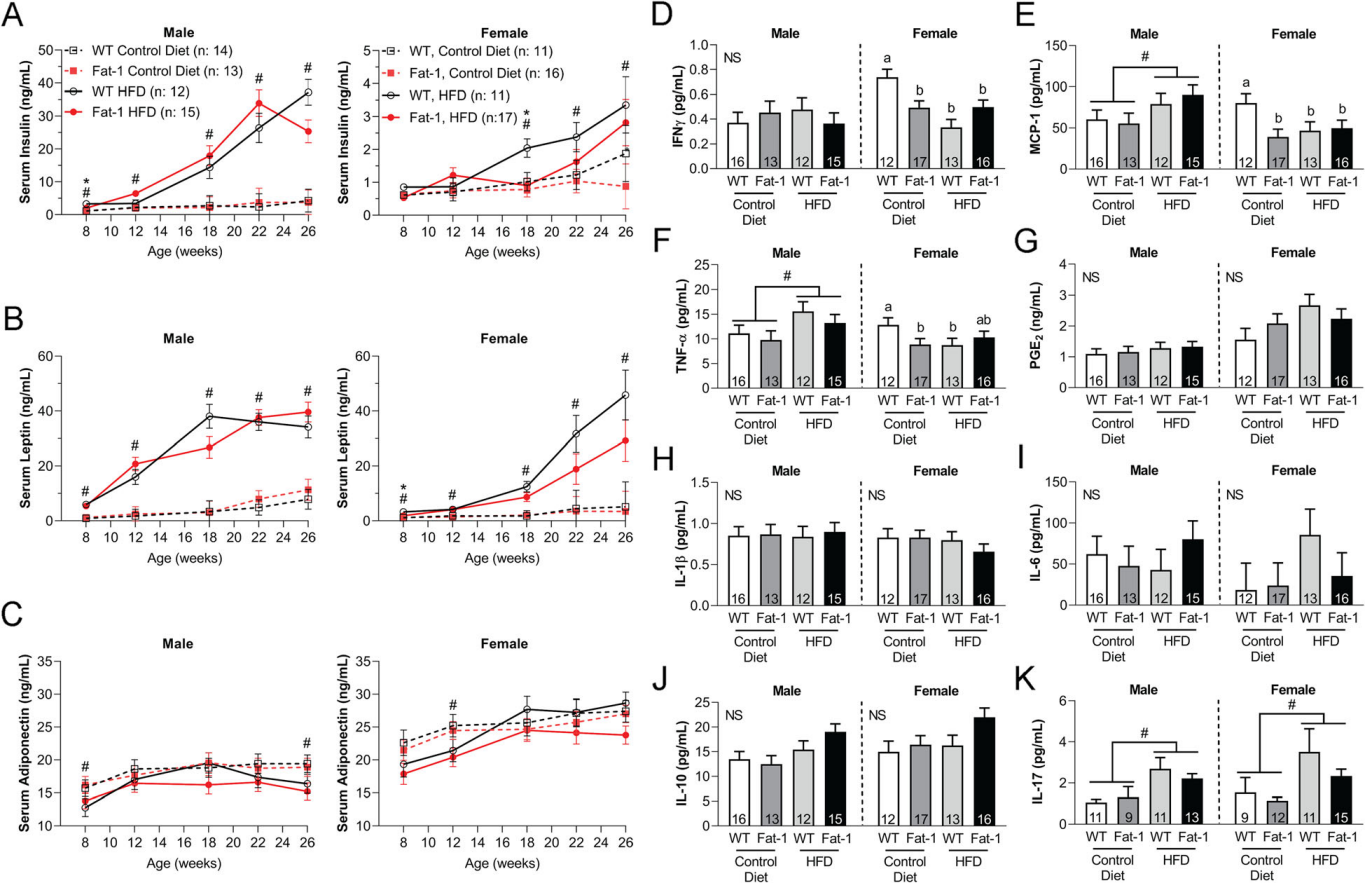
Serum levels of **a** insulin, **b** leptin, and **c** adiponectin over time. Serum cytokine levels at 28 weeks of age for **d** IFN-γ, **e** MCP-1, **f** TNF-α, **g** PGE_2_, **h** IL-1β, **i** IL-6, **j** IL-10, and **k** IL-17. ^#^*p* < 0.05, mice treated with HFD vs. mice treated with control diet within a specific time point. Two-way ANOVA within the same sex followed by Fisher’s LSD post hoc. NS, interaction term not significant (> 0.05). ^#^*p* < 0.05, diet effect. Groups not sharing the same letter are significantly different, *p* < 0.05. The number (*n*) of the mice used in each group is indicated on plots. Data presented as mean ± SEM

Male obese mice exhibited significantly higher serum insulin and leptin levels compared to lean male mice at 26 weeks of age regardless of the strain (Fig. 4a, b). Similar trends of increased serum insulin and leptin levels were also observed for obese female mice, although female obese *fat-1* mice showed relatively lower levels of insulin and leptin compared to female WT mice. Insulin levels for *fat-1* and WT male obese mice at later time points reached levels an order of magnitude above those of their lean male and both female counterparts. Obesity resulted in a moderate but significant decrease in serum adiponectin levels in male, but not female, mice at 26 weeks of age (Fig. 4c).

### Trends toward decreased pro-inflammatory cytokines and increased anti-inflammatory cytokines in *fat-1* transgenic mice

Serum cytokines were collected at 28 weeks of age (i.e., 12 weeks post-DMM surgery) and evaluated using a multiplex kit. Serum concentrations of pro-inflammatory cytokines including interferon-γ (IFN-γ), monocyte chemoattractant protein-1 (MCP-1), TNF-α, and IL-12p70 were either significantly decreased or trended lower in the female *fat-1* mice fed the control diet compared to female WT mice under the same diet (Fig. 4d–f; see supplementary Fig. S2). Obese female mice, regardless of strain, showed significantly lower serum levels of IFN-γ, MCP-1, and TNF-α compared to female WT fed the control diet. Male obese *fat-1* mice trended toward lower serum concentrations of IFN-γ and TNF-α versus male obese WT mice. Obese female *fat-1* mice also showed reduced serum prostaglandin E2 (PGE_2_), IL-1β, and IL-6 levels compared to obese female WT mice (Fig. 4g–i). Both male and female obese *fat-1* mice trended toward increased serum IL-10, but decreased serum IL-17 compared to the corresponding obese WT mice (Fig. 4j, k). Statistically significant increases in MCP-1 and TNF-α levels were seen in male obese mice as compared to their lean counterparts on control diet, and a similar increase in IL-17 levels was seen in both male and female mice. Synovial fluid from DMM-operated limbs showed statistically significant increases in IL-8 levels, as compared to non-operated contralateral control levels, in every group regardless of diet treatment, strain, or sex (see supplementary Fig. S3).

### Bivariate regression analysis between serum FAs, OA severity, and joint synovitis in *fat-1* mice and WT littermates demonstrated sex-dependent correlations

To evaluate the association between various lipid species with disease markers (i.e., OA and synovitis scores), WT and *fat-1* data for both HFD and control diets were pooled, but still separated by sex. Bivariate regression analysis was performed using lipid species as predictors. Correlation values for OA (modified Mankin scores) and synovitis scores show distinctly different results for male and for female mice (Table 1). Two ω-6 FAs (γ-linolenic acid and osbond acid) were positively associated with OA; docosanoic acid, a SFA, was positively associated with synovitis in male mice. Furthermore, eicosapentaenoic acid (EPA), an ω-3 FA, was negatively associated with OA severity. We also observed that nervonic acid, a MUFA, and all-cis-7,10,13,16,19-docosapentaenoic acid (DPA), an ω-3 FA, were both negatively associated with synovitis in male mice. Except for EPA (negatively associated with synovitis) for female mice, there were no statistically significant associations of FAs and OA or FAs and synovitis.

**Table 1:** Correlations between the normalized concentration (Mol%) of serum lipid species with OA severity and synovitis

		Male		Female	
Predictor variables		OA	Synovitis	OA	Synovitis
		***r***	***r***	***r***	***r***
**SFAs**	Myristic acid	− 0.20	− 0.23	0.13	0.17
	Pentadecylic acid	− 0.09	− 0.18	0.12	0.11
	Palmitic acid	0.18	− 0.01	− 0.10	0.09
	Stearic acid	− 0.25	− 0.02	− 0.06	− 0.16
	Docosanoic acid	0.00	0.45*	0.22	0.02
**MUFAs**	Myristoleic acid	− 0.21	− 0.30	0.08	0.14
	Palmitoleic acid	0.19	− 0.15	− 0.04	0.18
	Oleic acid	0.10	− 0.14	− 0.03	0.15
	Eicosenoic acid	− 0.16	− 0.07	− 0.09	0.15
	Nervonic acid	0.02	− 0.41*	0.09	− 0.02
**ω-6 FAs**	Linoleic acid	− 0.27	0.14	0.14	− 0.16
	γ-Linolenic acid	0.51**	0.18	0.04	− 0.27
	Eicosadienoic acid	0.11	0.23	0.12	0.01
	Arachidonic acid	0.12	0.07	− 0.02	0.02
	Osbond acid	0.56**	− 0.09	− 0.08	0.22
**ω-3 FAs**	α-Linolenic acid	− 0.13	− 0.22	0.05	− 0.06
	Stearidonic acid	− 0.14	− 0.29	− 0.12	− 0.01
	ETA	− 0.11	0.20	0.10	0.13
	EPA	− 0.39*	− 0.12	− 0.03	− 0.32*
	DPA	− 0.09	− 0.42*	− 0.15	− 0.20
	DHA	− 0.06	0.04	− 0.02	− 0.10
ω-6/ω-3 FA ratio		0.09	0.14	0.12	0.1

*ETA* eicosatetraenoic acid, *EPA* eicosapentaenoic acid, *DPA* all-cis-7,10,13,16,19-docosapentaenoic acid, *DHA* docosahexaenoic acid. *p* values less than 0.05 are shown in bold. *p < 0.05; **p < 0.01

### Multivariable regression analysis for variables predicting OA severity in male mice resulted in correlations with biochemical factors

To assess the relative contribution of biochemical and biomechanical factors (e.g., body weight) on OA severity, we constructed multivariate generalized linear models to identify which variables remained independently associated with the OA. Specifically, biochemical factors such as serum ω-3 FA (i.e., EPA), ω-6 FA (i.e., osbond acid), and leptin, along with biomechanical factors (i.e., body weight), were used to predict OA severity in male mice. Multivariable regression analysis was restricted to male mice due to general sex dependence of the associations between lipid species and OA. Biochemical factors, particularly serum EPA levels, but not body weight, were significantly associated with OA in male mice (Table 2).

**Table 2:** Multivariate regression analyses for variables predicting OA severity

Parameters	Model 1	Model 2
	***β***	***β***
**Biochemical**		
ω-6 FA (osbond acid)	0.36 (*p* < 0.08)	
ω-3 FA (EPA)	− 0.44*	
Leptin		− 0.24
**Biomechanical**		
Weight	− 0.15	− 0.06
**Whole model (*r***^***2***^**)**	0.47**	0.01

*β* standardized coefficient, *EPA* eicosapentaenoic acid. *p* values less than 0.05 are shown in bold. *p < 0.05; **p < 0.01

## Discussion

The findings of this study demonstrated that *fat-1* mice had decreased PTOA and joint synovial inflammation in a sex- and diet-dependent manner compared to WT mice, specifically with male *fat-1* mice showing a reduced Mankin OA score and female *fat-1* mice showing reduced synovitis relative to WT controls. The *fat-1* mice also had reduced serum ω-6/ω-3 FA ratios compared to their littermate WT mice. As all mice were fed the same diets in their specific diet groups, the difference in OA severity and synovitis between *fat-1* and WT mice can be mainly attributed to the increased levels of endogenous serum ω-3 FAs in *fat-1* mice.

While obese *fat-1* mice had decreased OA severity and decreased synovitis (female mice only) compared to the obese WT mice (sex-dependent significance), both groups had comparable body weight and percent body fat. These results indicate that altering circulating serum levels of pro- and anti-inflammatory FAs was sufficient to decrease obesity-associated OA, despite the fact that the body weight remained unchanged. Using multivariable regression analysis, we further demonstrated that OA severity was independent of body weight while exhibiting a negative correlation to serum levels of EPA in male mice. This observation is consistent with the results of our recent study showing that a “metabolic” factor such as systemic inflammation, rather than a “mechanical” factor such as body weight, is the main contributor of obesity-related OA following joint injury [17]. These findings are also in line with previous studies showing that leptin-deficient (*ob/ob*) and leptin receptor–deficient (*db/db*) transgenic mice, which naturally become obese on control chow diets, do not develop knee OA, further suggesting that body weight by itself may not be a risk factor for joint degeneration in obesity under a control diet [18].

In the present study, we observed that lean female *fat-1* mice had comparable OA severity compared to lean female WT mice post-DMM surgery. Malfait et al. showed sex-dependent differences in C57BL/6 mice 8 weeks following surgery, with female mice demonstrating reduced arthritic changes compared to male mice [19]. However, with obesity, female mice demonstrated increased OA [20]. In this study, we observed trends toward mitigated OA scores in obese female *fat-1* mice. It has been recently reported that when fed a control diet, *fat-1* mice showed no differences in idiopathic OA compared to WT mice [15]. These results imply that the beneficial effects of ω-3 FAs for preventing OA may be accentuated in trauma- and/or diet-induced obesity. Nonetheless, Huang et al. reported that lean female *fat-1* mice had significantly lower OA scores compared to lean female WT mice following joint injury [16]. The discrepancy between their study and ours may result from the different time points when the joints were harvested: while the authors of the previous study harvested the joints at 8 weeks following DMM surgery, we sacrificed the mice 12 weeks post-injury. Collectively, these results suggest that increased levels of serum ω-3 FAs delayed OA onset in lean female mice at early time points. However, the severity of cartilage degradation likely reached the same levels at later time points for both female *fat-1* and WT mice, supporting the notion that anti-catabolic effects of ω-3 FA on PTOA is more prominent in obesity.

Adipokines, such as leptin and adiponectin, as well as other circulating factors such as insulin are associated with “metabolic syndrome” and have been extensively studied in obesity-linked OA [17, 21]. For example, leptin and insulin levels were elevated in mice on an ω-6 FA-rich HFD compared to those on an ω-3 FA-rich HFD, and leptin had a positive association with OA severity [17]. In the current study, a significantly lower serum leptin concentration was observed in obese female *fat-1* mice at 26 weeks of age compared to obese WT mice, which is consistent with previous studies showing that *fat-1* mice on a HFD had significantly lower levels of leptin compared to their WT littermates [22]. Although a number of serum and synovial fluid cytokines analyzed did not reach statistical significance by diet or strain, we did observe that lean female *fat-1* mice exhibited decreases in serum pro-inflammatory cytokines, including IFN-γ, TNF-α, and MCP-1, compared to lean female WT mice, providing a potential mechanism for the reduction in joint synovitis. Our observation of decreased serum IFN-γ and MCP-1 levels in lean female *fat-1* mice is in line with the results of a recent study showing that *fat-1* mice exhibit lower gene expression of these two inflammatory molecules in the ankle tissues compared to WT mice after induction of rheumatoid arthritis [23].

Several putative mechanisms through which ω-6 and ω-3 FAs modulate inflammation have been proposed [24–26]. For example, arachidonic acid, an ω-6 FA, can be converted into PGE_2_, which may play a role in enhancing IL-1β–associated cartilage degradation [27]. On the contrary, ω-3 FAs can enhance insulin sensitivity through G protein–coupled receptor 120 (GPR120) [28], while GRP120 knock-out mice showed accelerated cartilage degeneration compared to WT mice [29]. In the current study, serum ω-6 FAs, particularly γ-linolenic acid and osbond acid, were positively associated with OA in male mice, while serum ω-3 FAs, such as EPA and DPA, were negatively associated with OA and synovitis, respectively. These results corroborate our previous studies showing that obesity-associated OA had opposite associations to serum levels of γ-linolenic acid and EPA in mice. Moreover, serum ω-6 FAs were positively associated with knee synovitis in humans [30]. Growing evidence suggests that dietary FAs can significantly influence circulating levels of various cytokines and adipokines associated with obesity and metabolic syndrome [11–13]. Collectively, our findings provide further evidence that ω-6 FAs play a role as pro-inflammatory mediators, whereas ω-3 FAs may act in an anti-inflammatory manner in obesity and obesity-associated OA.

Both WT and *fat-1* mice exhibited similar serum levels of SFAs and MUFAs in molar percentage (mole %), although obese mice fed HFD had decreased serum levels of MUFAs compared to lean mice receiving the control diet. This result indicates that altering ω-6/ω-3 FA ratios has a minimal effect, if any, on the metabolic processes of other types of FAs. Additionally, we found that serum levels of docosanoic acid had a positive correlation with synovitis in male mice. Despite this positive association, it remains unclear how docosanoic acid modulates joint synovitis, although exposure of oligodendrocytes and astrocytes to docosanoic acids led to cell death by inducing mitochondrial depolarization [31]. The observation of the negative association between serum levels of nervonic acid with synovitis in male mice is consistent with our previous findings showing that obese male mice fed ω-3 FA-rich HFD exhibited decreased synovitis and had higher levels of serum nervonic acid compared to the obese mice treated with HFD rich in ω-6 FA or SFA [32], implying a potential anti-inflammatory effect of nervonic acid on synovial inflammation.

We did not observe strong correlations between FAs and OA in female mice; however, serum EPA concentration was negatively associated with joint synovitis. This may be attributed to the generally lower OA scores in female mice compared to male mice, suggesting sex-dependent cartilage degeneration in response to HFD and injury. This result is consistent with a study showing that sex hormones play a critical role in the pathogenesis of injury-induced OA as male mice had more severe OA than females following DMM surgery, while female mice with ovariectomy had exacerbated OA progression due to loss of sex hormones [33]. We also observed that serum insulin concentrations were 10-fold lower in female mice compared to males and that female mice had less weight gain versus male mice under high-fat feeding. In our previous study, female C57BL/6J mice also showed variation in susceptibility to diet-induced obesity [34]. Recently, it has been reported that increases in a regulatory T cell population, instead of inflammatory macrophages, in the adipose tissue upon HFD may help female mice resist obesity-associated inflammation [35], providing a plausible explanation for the observation of low OA in female mice in our study. Additionally, previous findings indicate that estrogen levels are linked to joint homeostasis [36], and estrogen may regulate leptin secretion [37], suggesting potential mechanisms for the sex differences observed in the current study. Nevertheless, future studies are required to elucidate the mechanisms behind the sex differences in obesity-related inflammation and OA development.

## Conclusions

In summary, we demonstrated that *fat-1* mice exhibited decreased serum ω-6/ω-3 FA ratios, resulting in a mitigation of OA severity despite exhibiting similar body weight compared to WT mice. Interestingly, *fat-1* male mice did not exhibit a reduction in synovitis or osteophyte scores, suggesting that different mechanisms are involved in regulating degenerative changes in the cartilage and joint structure, as compared to those regulating synovitis. Our findings provide further evidence that the role of circulating FA composition and associated changes in systemic metabolic inflammation, rather than body weight, may be the major risk factor for obesity-associated OA. Furthermore, the current study also indicates the potential therapeutic benefit of decreasing the serum ω-6/ω-3 FA ratio for mitigating arthritic changes in obese individuals.

## Supplementary information

**Additional file 1.** Supplementary methods and figures

## Data Availability

The datasets generated and/or analyzed during the current study are not publicly available due to the limit of storage space but are available from the corresponding author on reasonable request.

## Abbreviations

OA: Osteoarthritis
PTOA: Post-traumatic osteoarthritis
DMM: Destabilization of the medial meniscus
WT: Wild type
HFD: High-fat diet
FA: Fatty acid
ω-3 FA: Omega-3 fatty acid
ω-6 FA: Omega-6 fatty acid
SFA: Saturated fatty acid
PUFA: Polyunsaturated fatty acid
MUFA: Monounsaturated fatty acid
ETA: Eicosatetraenoic acid
EPA: Eicosapentaenoic acid
DPA: Docosapentaenoic acid
DHA: Docosahexaenoic acid
IL-1: Interleukin 1
IL-6: Interleukin 6
IL-10: Interleukin 10
IL-17: Interleukin 17
IL-12p70: Interleukin-12 heterodimer
IFN-γ: Interferon gamma
TNF-α: Tumor necrosis factor alpha
MCP-1: Monocyte chemoattractant protein
PGE_2_: Prostaglandin E2
GPR120: G protein–coupled receptor 120
DXA: Dual-energy X-ray absorptiometry
BMD: Bone mineral density
