# Supplementary material for: Transgenic conversion of ω-6 to ω-3 polyunsaturated fatty acids via fat-1 reduces the severity of post-traumatic osteoarthritis
Source: Arthritis Res Ther. 2020 Apr 15;22:83. doi: 10.1186/s13075-020-02170-7 (PMC7160898; doi:10.1186/s13075-020-02170-7)
Supplement: Supplementary file 1 — Additional file 1. Supplementary methods and figures [file 13075_2020_2170_MOESM1_ESM.docx]

**Supplementary material**

**Transgenic conversion of ω-6 to ω-3 polyunsaturated fatty acids via *fat-1* reduces the severity of post-traumatic osteoarthritis**

Kelly A. Kimmerling^1,2^, Sara J. Oswald^1,2^, Janet L. Huebner^3^, Dianne Little^4^, Virginia B. Kraus^3,5^, Jing X. Kang^6^, Chia-Lung Wu^1,2,†^, Farshid Guilak^1,2,†^

^1^Department of Orthopaedic Surgery, Washington University, St. Louis, St. Louis, MO, USA.

^2^Shriners Hospital for Children - St. Louis, St. Louis, MO, USA.

^3^Duke Molecular Physiology Institute, ^5^Department of Medicine, Duke University School of Medicine, Durham, NC, USA.

^4^Departments of Basic Medical Sciences and Biomedical Engineering, Purdue University, West Lafayette, IN, USA.
^6^ Laboratory for Lipid Medicine and Technology, Department of Medicine, Massachusetts General Hospital and Harvard Medical School, Boston, MA, USA.

^†^These authors contributed equally.

Correspondence address:

Farshid Guilak, Ph.D.

Washington University in St. Louis

Campus Box 8233

McKinley Research Bldg, Room 3121

St. Louis, MO 63110

USA

Email: [guilak@wustl.edu](mailto:guilak@wustl.edu)

Chia-Lung Wu, Ph.D.

Washington University in St. Louis

Campus Box 8233

McKinley Research Bldg, Room 3121

St. Louis, MO 63110

USA

Email: [chia-lung.wu@wustl.edu](mailto:chia-lung.wu@wustl.edu)

**SUPPLEMENTARY METHODS**

**Animals, breeding, and study groups.**  All animal procedures were performed using a Duke University Institutional Animal Care and Use Committee-approved protocol. Two *fat-1* breeding pairs were used to generate all mice for this study. C57BL/6-fat-1 littermates were separated by sex, weaned, chipped, and tail snipped (≤ 5mm) for genotyping (Transnetyx) at 3 weeks of age. Four overall factors were examined, depending on the analysis: strain (WT versus *fat-1*), sex (male versus female), diet (control low-fat diet [Control Diet] versus high-fat [HFD]), and surgery (left limb [DMM] versus right limb [non-operated] control).

**Weight tracking and diets.** Custom diets were supplied by Research Diets, Inc. (New Brunswick, NJ), and consisted of an ω-6 rich HFD (60% kcal from fat, D11120105) and its corresponding Control Diet (10% kcal from fat, D11120103). All diets were kept at -20°C until use and animals were fed *ad libitum*.

**Longitudinal collection of serum.**  Maxillary venipuncture from each mouse at weeks 8, 12, 18, 22, and 26 was used for blood collection. Blood collections were performed by a trained veterinary technician using a sterile lancet and collection tubes with ~100 µL of blood collected per stick. No more than 10% of total blood volume was collected from each animal at each timepoint. Serum was isolated, aliquoted, and stored at -80°C until analysis.

**DXA scanning of mice.** Body fat percentage and bone mineral density of mice were obtained using a dual-energy x-ray absorptiometry (DXA) scanner (GE Lunar PIXImus, GE Healthcare, Madison, WI, USA) at weeks 10, 14, and 28. The DXA machine was calibrated for bone mineral density and percent fat prior to scanning. Mice were anesthetized using isoflurane, placed ventral side down to capture the entire body and tail, and scanned. Data were analyzed using the PIXImus2 software. The head region was excluded from all analyses. Final values included the body bone mineral density (BMD) and the percent fat (fat content/total tissue mass).

**Destabilization of the medial meniscus (DMM) model.** At 16 weeks of age, mice underwent DMM surgery in the left hind limb to induce PTOA. Mice were anesthetized using isoflurane and positioned on a custom cradle with the knee at 90° flexion during surgery. An incision through the joint was made, and dissection continued through the medial third of the patellar tendon and the fat pad. The medial meniscotibial ligament was then sectioned using a #11 blade, and medial displacement of the medial meniscus beyond its normal range was confirmed. The joint capsule and subcutaneous skin layer were sutured using a continuous 8-0 suture (Polysorb^TM^, Covidien, Mansfield, MA, USA), and skin glue was used to close the skin. Buprenorphine was given daily post-surgery for 72 hours. The right hind limb served as an unoperated contralateral control limb within each mouse.

**Collection of materials at sacrifice.** Mice from each group were sacrificed 12 weeks post-surgery (28 weeks old). Serum and synovial fluid from each limb were collected from each mouse and stored for use in biomarker analyses as described previously [1].

**Histological processing of limbs.** Both hind limbs from each mouse were harvested and fixed in 10% neutral buffered formalin for 48 hours. Decalcification of the limbs was accomplished using Cal-Ex Decalcification Solution (Fisher Scientific, Pittsburgh, PA) for 5 days. Limbs were then dehydrated, and paraffin-embedded using the Leica ASP300S Tissue Processor (Leica Microsystems, Buffalo Grove, IL). Serial coronal plane sections of 8μm were taken through each joint for assessment of articular cartilage degeneration, synovitis, and osteophyte formation.

**Articular cartilage assessment.** Slides were stained with Harris Hematoxylin, Fast Green (aqueous), and Safranin-O in order to assess articular cartilage degeneration. A modified Mankin score was used by four blinded graders to determine OA severity for each quadrant (lateral femoral condyle, lateral tibial plateau, medial femoral condyle, medial tibial plateau) [2, 3]. Joint quadrant scores were then tabulated and averaged between graders to get an overall total joint score of articular cartilage degeneration for each limb.

**Synovial inflammation assessment.** Slides were stained with Harris Hematoxylin and Eosin in order to assess the level of synovitis. A modified Krenn score was used by four blinded graders to determine synovitis severity for each quadrant (lateral femoral condyle, lateral tibial plateau, medial femoral condyle, medial tibial plateau) [4, 5]. Joint quadrant scores were then tabulated and averaged between graders to get an overall total joint score of synovitis for each limb.

**Osteophyte assessment.** Slides were stained with Harris Hematoxylin, Fast Green (aqueous), and Safranin-O in order to assess osteophyte development. A modified quantitative score [6] was used by four blinded graders to determine the maturity of osteophytes present for each quadrant (lateral femoral condyle, lateral tibial plateau, medial femoral condyle, medial tibial plateau). Osteophytes were graded under a 100x magnification. The scale was as follows: grade 0-origin (normal surface), grade 1-early chondrophyte (fibrous outgrowths), grade 2-fibrocartilage/chondrophytes (fibrous/cartilaginous tissue; no hypertrophic chondrocytes or bone formation present), grade 3-early osteophyte (zonal pattern of chondrocytes with columnar alignment; deepest cells show hypertrophy), grade 4-mature osteophyte (ossification; tissue resembles articular cartilage; hypertrophic chondrocytes; no clear tidemark). Joint quadrant scores were then tabulated and averaged between graders to get an overall total joint score of osteophyte development for each limb.

**Serum and synovial fluid biomarker assays.** Serial serum was collected at 8, 12, 18, 22, and 26 weeks of age, while serum and synovial fluid were collected at sacrifice (28 weeks) for use in biomarker analyses. The Mouse Metabolic Kit (Meso Scale Diagnostics, Rockville, MD), the Mouse Adiponectin Kit (Meso Scale Diagnostics, Rockville, MD), and the Mouse IL-17 Kit (Meso Scale Diagnostics, Rockville, MD) were used to analyze serial serum collections from 8 weeks to sacrifice. The Mouse Metabolic Kit measures both insulin and leptin, while the other two kits measure adiponectin and IL-17, respectively. For the Mouse Metabolic Kit, the samples were run undiluted using 10μL as directed by the manufacturer. For this kit, the reported mean intra- and inter-assay coefficients of variation of the leptin assay were 3.9% and 10.9%; the minimum detectable concentration was reported to be 43pg/mL. For insulin, the reported mean intra- and inter-assay coefficients of variation were 4.2% and 8.4%, and the minimum detectable concentration was reported to be 15pg/mL. For the Mouse Adiponectin Kit, samples were run diluted 1:1000 using 5µL as directed by the manufacturer. For this kit, the reported intra- and inter-assay coefficients of variation for adiponectin were 3.8% and 20.2%; the minimum detectable concentration was reported to be 0.04ng/mL. For the Mouse IL-17 kit, the samples were run diluted at 1:2 if volume was limited, or undiluted if 25μL of volume (as recommended by the manufacturer) was available. For this kit, the intra- and inter-assay coefficients of variation for IL-17 were 5.7% and 7.0%, and the minimum detectable concentration was reported to be 5.5pg/mL.

The Mouse Pro-Inflammatory Panel 1 Kit (Meso Scale Diagnostics, Rockville, MD) was used to measure serum and synovial fluid concentrations of 10 cytokines in samples acquired at the time of sacrifice, including interferon-gamma (IFN-γ), interleukin-1 beta (IL-1β), IL-2, IL-4, IL-5, IL-6, IL-8/keratinocyte-derived protein chemokine (KC), IL-10, IL-12p70, and tumor necrosis factor alpha (TNF-α). All samples were run at a dilution of 1:2, as directed by the manufacturer, and required 25µL for the assay. The mean intra-assay CV within a plate and inter-assay CV between plates were calculated and tabulated (**supplementary Table S1).** For measured synovial fluid cytokine concentration, every analyte, except KC/IL-8, had ≥ 77% of samples below the LLOD and thus were excluded from analysis.

**Lipidomics assays.** Serum samples were analyzed using the Fatty Acid Metabolism Panel (Metabolon). Thirty-three different targets were measured, including 7 saturated FAs, 21 unsaturated FAs, and 5 additional phospholipid-derived fatty acids. To obtain ratios, all ω-6 FAs and ω-3 FAs were individually summed; the concentration of ω-6 FAs was then divided by the concentration of ω-3 FAs to obtain the ratio. The lipid species used for bivariate and multivariate regression analyses are listed in **supplementary Table S2.**

**Statistical analysis.** All results were analyzed using STATISTICA (v.13, Dell Inc., Tulsa, OK) with significance reported at the 95% confidence level. Detailed statistical methods are provided in the caption of each figure.

**SUPPLEMENTARY TABLES**

**Table S1. Mean intra- and inter-assay coefficients of variation (CV)**

| **Analyte** | **Mean Intra-assay CV (within-plate)  based on STDs** | **Reported Intra-assay CV (within plate) based on STDs** | **Reported Intra-assay CV (within plate) based on control samples** | **Mean Inter-assay  CV (between plates) based on STDs Excluding zero** | **Reported Inter-assay CV (between plates)** |
| --- | --- | --- | --- | --- | --- |
| IFN-γ | 5.1% | 6.3% | 3.0% | 1.6% | 11% |
| IL-1β | 4.9% | 6.1% | 2.4% | 2.8% | 10.5% |
| IL-2 | 6.8% | 7.0% | 2.7% | 3.4% | 10.5% |
| IL-4 | 5.2% | 6.4% | 2.7% | 2.4% | 10% |
| IL-5 | 4.6% | 7.1% | 2.7% | 1.7% | 14.5% |
| IL-6 | 5.2% | 5.2% | 2.5% | 3.9% | 10.7% |
| KC/IL-8 | 6.1% | 6.9% | 2.3% | 3.0% | 10.3% |
| IL-10 | 6.1% | 8.6% | 4.1% | 5.4% | 10.3% |
| IL-12p70 | 8.6% | 7.3% | 2.1% | 10.2% | 11.8% |
| TNF-α | 3.2% | 6.5% | 2.5% | 6.2% | 11.6% |

**Table S2. Lipid species investigated in the current study**

|  | |
| --- | --- |
| **SFAs** | myristic acid (14:0) |
|  | pentadecylic acid (15:0) |
|  | palmitic acid (16:0) |
|  | stearic acid (18:0) |
|  | docosanoic acid (22:0) |
| **MUFAs** | myristoleic acid (14:1n5) |
|  | palmitoleic acid (16:1n7) |
|  | oleic acid (18:1n9) |
|  | eicosenoic acid (20:1n9) |
|  | nervonic acid (24:1n9) |
| **ω-6 PUFAs** | linoleic acid (18:2n6) |
|  | γ-linolenic acid (18:3n6) |
|  | eicosadienoic acid (20:2n6) |
|  | arachidonic acid (20:4n6) |
|  | osbond acid (22:5n6) |
| **ω-3 PUFAs** | α-linolenic acid (18:3n3) |
|  | stearidonic acid (18:4n3) |
|  | ETA (20:4n3) |
|  | EPA (20:5n3) |
|  | DPA (22:5n3) |
|  | DHA (22:6n3) |
| Osbond acid: all-*cis*-4,7,10,13,16-docosapentaenoic acid; ETA: eicosatetraenoic acid; EPA: eicosapentaenoic acid; DPA: all-cis-7,10,13,16,19-docosapentaenoic acid; DHA: docosahexaenoic acid | |

**SUPPLEMENTARY FIGURES**

**Fig. S1**

**
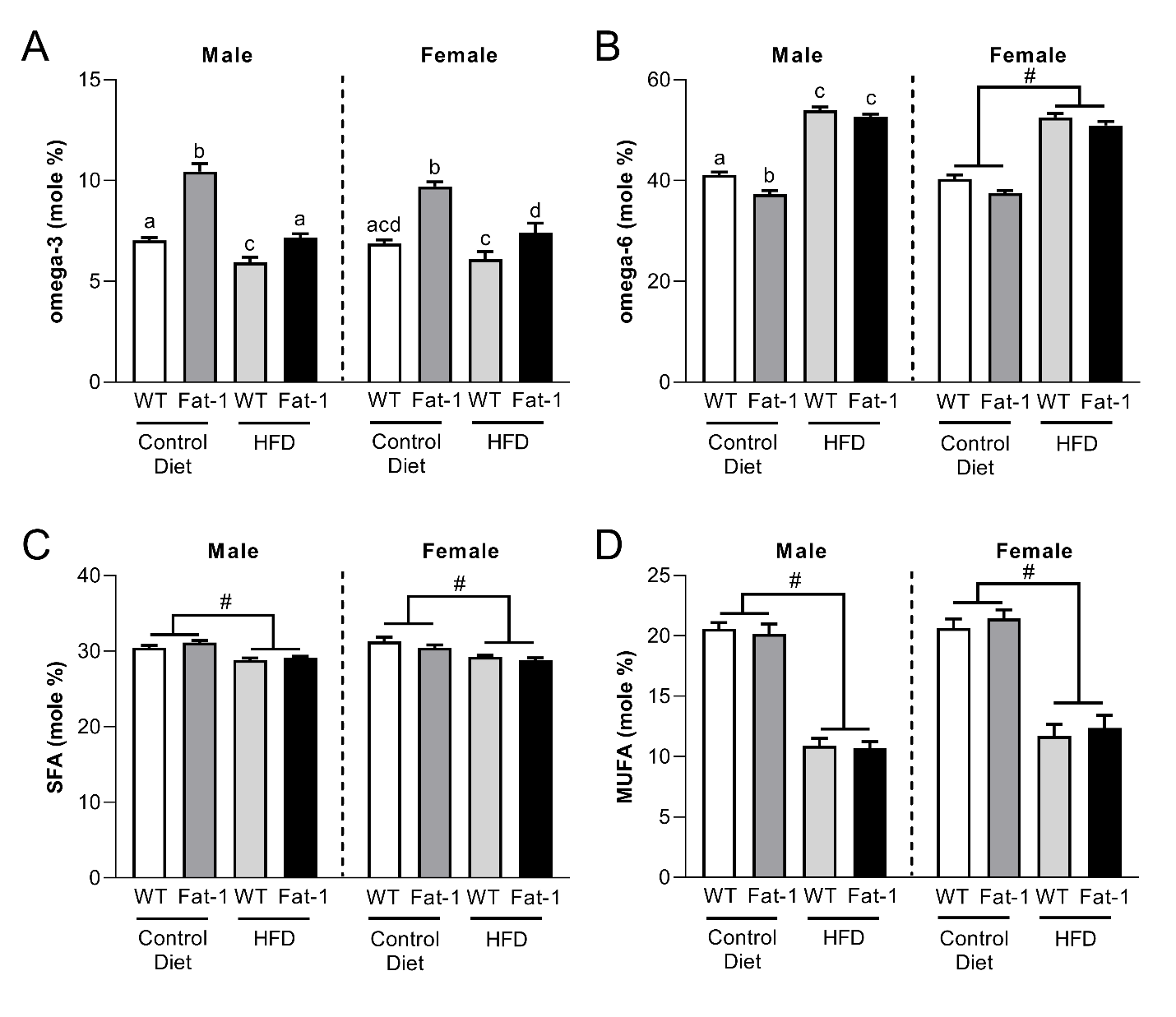
**

**Fig. S1.** *Fat-1* mice exhibited significantly higher levels of (**A**) ω-3 molar percentage of total FAs (mole %), FA, while both control diet WT and *fat-1* mice exhibited similar serum levels of (**B**) mole % of ω-6 FA, (**C**) saturated fatty acids (SFAs), and (**D**) monounsaturated fatty acids (MUFAs). Two-way ANOVA followed by Fisher’s LSD post-hoc. Groups not sharing the same letter are significantly different, p < 0.05. # p < 0.05, HFD *fat-1* vs. WT DMM-operated joints. Data presented as mean ± SEM. Male Control Diet WT n=16, *fat-1* n=13. Male HFD WT n=12, *fat-1* n=15. Female Control Diet WT n=12, *fat-1* n=17. Female HFD WT n=13, *fat-1* n=16.

**Fig. S2**

**
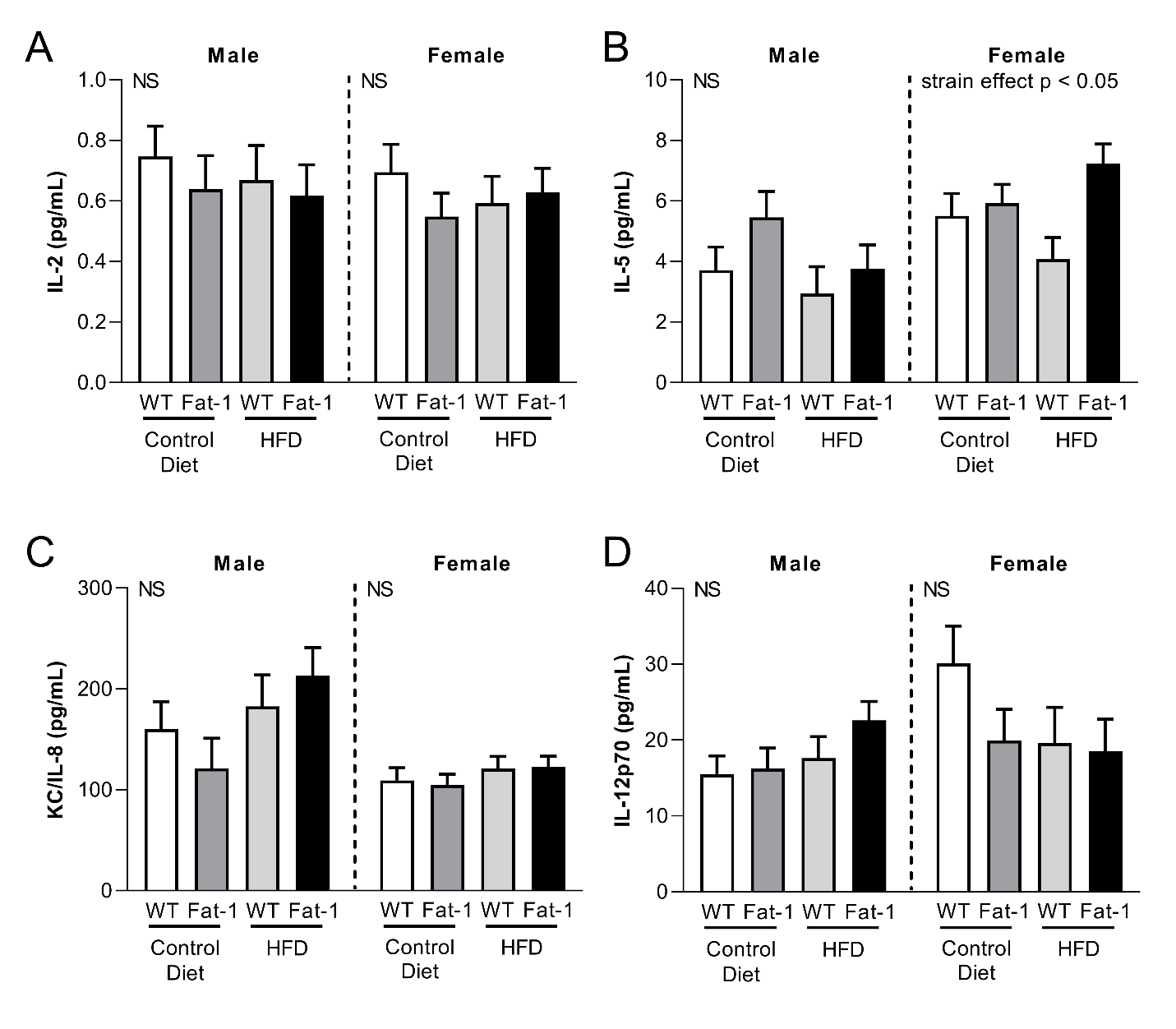
**

**Fig. S2.** Serum cytokine levels including (**A**) IL-2, (**B**) IL-5, (**C**) IL-8, and (**D**) IL-12p70 at 28 weeks of age. Two-way ANOVA within the same sex. NS: interaction term not significant (> 0.05). Data presented as mean ± SEM. Male Control Diet: WT n=16, *fat-1* n=13. Male HFD: WT n=12, *fat-1* n=15. Female Control Diet: WT n=12, *fat-1* n=17. Female HFD: WT n=13, *fat-1* n=16.

**Fig. S3**

**
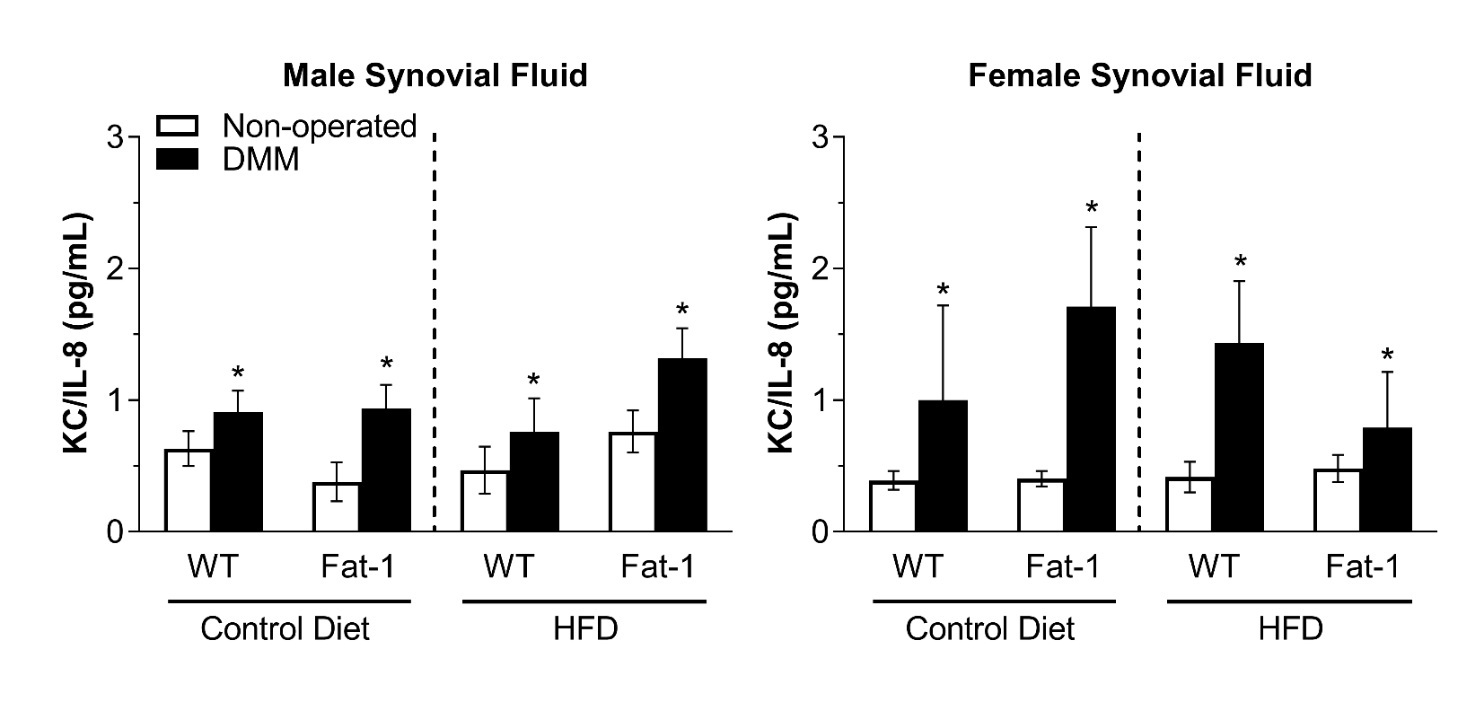
**

**Fig. S3.**  IL-8 levels in the synovial fluid of the operated and non-operated joints at 28 weeks of age. Two-way repeated measures ANOVA within the same sex followed by Fisher’s LSD post-hoc. * p < 0.05, DMM-operated vs. non-operated joints. Data presented as mean ± SEM.

**REFERENCES**

1. Seifer DR, Furman BD, Guilak F, Olson SA, Brooks SC, 3rd, Kraus VB. Novel synovial fluid recovery method allows for quantification of a marker of arthritis in mice. Osteoarthritis Cartilage. 2008;16(12):1532-8.

2. Mankin HJ, Lippiello L. The glycosaminoglycans of normal and arthritic cartilage. J Clin Invest. 1971;50(8):1712-9.

3. Furman BD, Strand J, Hembree WC, Ward BD, Guilak F, Olson SA. Joint degeneration following closed intraarticular fracture in the mouse knee: a model of posttraumatic arthritis. J Orthop Res. 2007;25(5):578-92.

4. Krenn V, Morawietz L, Burmester GR, Kinne RW, Mueller-Ladner U, Muller B, et al. Synovitis score: discrimination between chronic low-grade and high-grade synovitis. Histopathology. 2006;49(4):358-64.

5. Lewis JS, Jr., Furman BD, Zeitler E, Huebner JL, Kraus VB, Guilak F, et al. Genetic and cellular evidence of decreased inflammation associated with reduced incidence of posttraumatic arthritis in MRL/MpJ mice. Arthritis Rheum. 2013;65(3):660-70.

6. van Osch GJ, van der Kraan PM, van Valburg AA, van den Berg WB. The relation between cartilage damage and osteophyte size in a murine model for osteoarthritis in the knee. Rheumatol Int. 1996;16(3):115-9.
